# Orofacial fascial space abscess disguised as temporomandibular disorder: a report of 3 cases and literature review

**DOI:** 10.1186/s12903-023-03800-7

**Published:** 2024-01-03

**Authors:** Tae-Seok Kim, Yeon-Hee Lee

**Affiliations:** Department of Orofacial Pain and Oral Medicine, Kyung Hee University Dental Hospital, #26 Kyunghee-daero, Dongdaemun-gu, Seoul, 02447 South Korea

**Keywords:** Orofacial fascial space abscess, Temporomandibular disorder, Diagnosis, Infection, Computed tomography, Magnetic resonance imaging

## Abstract

Fascial space abscess is a condition in which infections spread into fascial spaces. It is a severe and life-threatening disease unless treated at an early stage. Due to the similarity of clinical symptoms, fascial space abscesses in the orofacial area are often disguised as other diseases, such as temporomandibular disorder (TMD). In this case series, we report three cases of fascial space abscesses disguised as TMD. In all cases, patients complained of severely limited mouth opening and pain in the temporomandibular joint (TMJ) and masseter muscles, which led clinicians to diagnose them with TMD. After two patients showed facial swelling and the third complained of dyspnea, clinicians realized the possibility of an orofacial fascial space abscess. On further evaluation, all patients showed increased C-reactive protein in blood tests, and the location of the fascial space abscess was confirmed by enhanced computed tomography images. Moreover, all patients had suspicious sources of odontogenic infections in panoramic images, periapical abscess on maxillary molars and periodontal disease on maxillary and mandibular molars, which were not appropriately evaluated at the first visit. This case series emphasizes the need for clinicians to realize the possibility of orofacial fascial space abscesses based on: clinical symptoms of severely limited mouth opening (< 15 mm) with pain in the facial area, including TMJ or masseter muscle, and possible sources of infection such as odontogenic infection, other infectious lesions, trauma, or invasive treatments. These clinical insights will enable the early detection of fascial space abscesses.

## Background

Fascial spaces are the spaces between the various layers of muscles, filled with loose connective tissue [1]. In the orofacial area, major fascial spaces include submandibular, sublingual, infratemporal, canine, buccal, and masticator spaces. Among those, masticator spaces are divided into masseteric, pterygoid, and temporal space. Fascial spaces are effective barriers, but they also act as the pathway for progressing infection. In the orofacial area, the most common source of infection is an odontogenic infection, which accounts for about 78% of space abscesses [2]. Other possible sources of infection include trauma, sinus disease, and invasive treatment of the orofacial area [3]. Common symptoms of fascial space infection and abscess in the orofacial area include limited mouth opening, neck stiffness, dyspnea, dysphagia, fever, swelling and redness [4]. If the diagnosis and treatments of fascial space abscess in the orofacial area are delayed, the abscess can spread into more critical structures including neck, airway, orbital septum, and even brain, leading to grave consequences [5, 6].

Although early detection and treatment are necessary to prevent the progress of infection, diagnosis of fascial space abscess in the orofacial area is often challenging. Conventional images, such as panoramic images, have limitations in detecting an infectious state of soft tissues. Although clinical examinations might help identify typical symptoms of fascial space abscesses, early fascial abscesses are often disguised as other diseases due to their ambiguous symptoms. One of the diseases that clinicians might confuse with orofacial fascial space abscess is temporomandibular disorder (TMD), especially when only limited mouth opening and pain are observed [7]. TMD is a multidisciplinary disorder and related to multiple causes such as psychology, general health, or spinal disorders, and thus it is common for any other orofacial disease including fascial space abscess to mimic TMD [8]. Since TMD and space abscess treatments are quite different, clinicians need to differentiate TMD and space abscess as early as possible. We herein report three cases in which patients were diagnosed with TMD at first visit but finally proven to have fascial space abscess by additional symptoms and further diagnosis.

## Case presentation

This case research was approved by the Institutional Review Board of the Kyung Hee University Dental Hospital (IRB no. KH-DT23014). Informed consent was obtained from all three patients. *Informed consent was obtained* from *all participants* in *the* study.

### Case 1

**Fig. 1:**
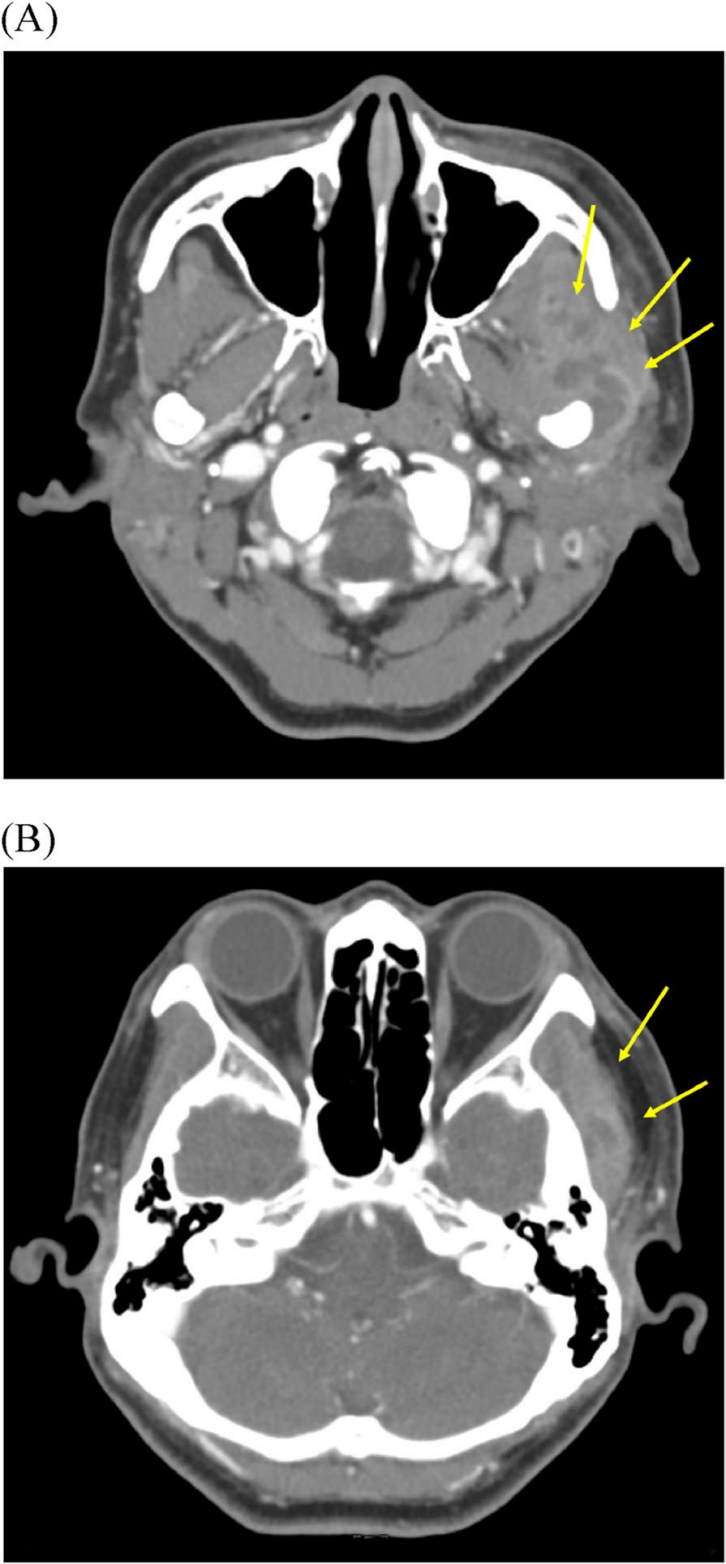
Enhanced CT images at first visit (case 1). Heterogeneous lesions of left masseteric and temporal space (yellow arrows) were observed. **A** Masseteric space. **B** Temporal space

**Fig. 2:**
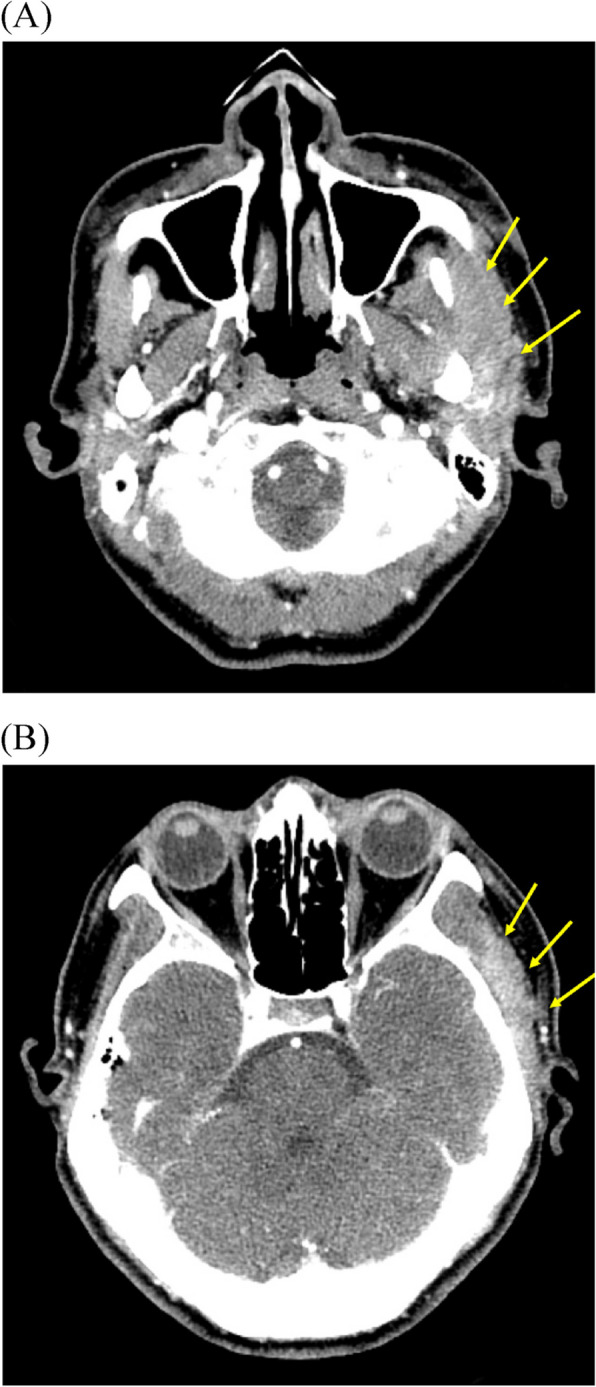
Neck angiography CT images 2 months after the first visit (case 1). Heterogeneous lesions of left masseteric and temporal space (yellow arrows) were decreased but remained. **A** Masseteric space. **B** Temporal space

A 69-year-old female patient from Seoul, Republic of Korea, visited the Department of Orofacial Pain and Oral Medicine at the Kyung Hee University Dental Hospital with the chief complaints of limited mouth opening, severe pain, and swelling of the left temporomandibular joint (TMJ). She had visited another clinic 2 months ago and was diagnosed with TMD of the left TMJ. However, her symptoms had worsened the day before she visited our clinic. During clinical examinations, her maximum mouth opening amount was only 15 mm, with pain in the left TMJ during mouth opening. During the lateral movement of the mandible, her range was severely limited to just 1 mm in both the right and left directions. Slight swelling and local heat were observed in the left TMJ and masseter. In the panoramic image, a periapical lesion with alveolar bone loss was observed in the #26 tooth. Although she had already been diagnosed with TMD, we suspected that there might be an infectious lesion. We decided to have an enhanced computed tomography (CT) and blood test for further evaluation. Signs of infection were observed in blood tests: white blood cell (WBC) 13,080/μL, erythrocyte sedimentation rate (ESR) 120 mm/hour, C-reactive protein (CRP) 5.33 mg/dL. In the enhanced CT scan, abscess and myositis were observed in the masticator space, and the suspected source of infection was a periapical lesion around the #26 tooth (Fig. 1). Final diagnosis was masticator space abscess. We prescribed antibiotics, and her symptoms improved. However, 2 months after the first visit, she complained of severely limited mouth opening and pain again. We decided to refer to the Department of Oral and Maxillofacial Surgery for further treatment. She was admitted to the ward and performed neck angiography CT. Since there were still abscess lesions (Fig. 2), she had supportive care, including intravenous antibiotics for 5 days. After administration, her symptoms improved, and the maximum mouth-opening amount was increased to 28 mm. We will keep regular follow-up checks.

### Case 2

**Fig. 3:**
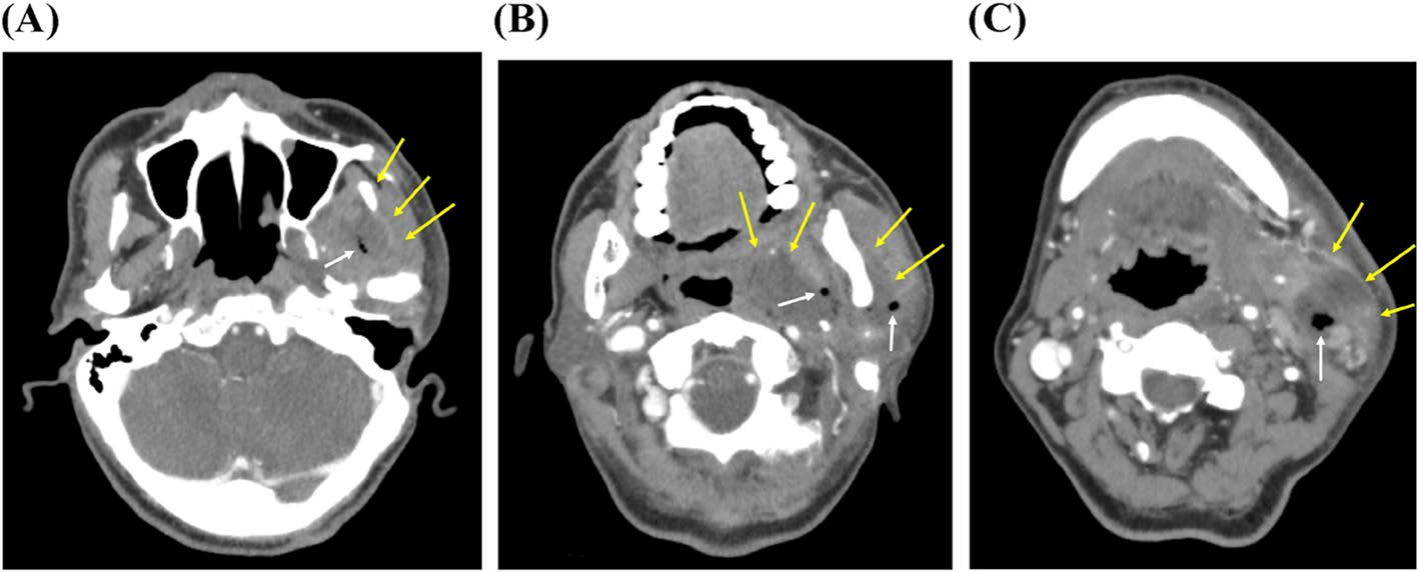
Neck angiography CT images 5 days after the first visit (case 2). Extensive heterogeneous lesions of the fascial space and muscles (yellow arrows) with inner aerobic voids (white arrows) were observed. **A** Masseteric space. **B** Parapharyngeal and masseteric space. **C** Submandibular space

**Fig. 4:**
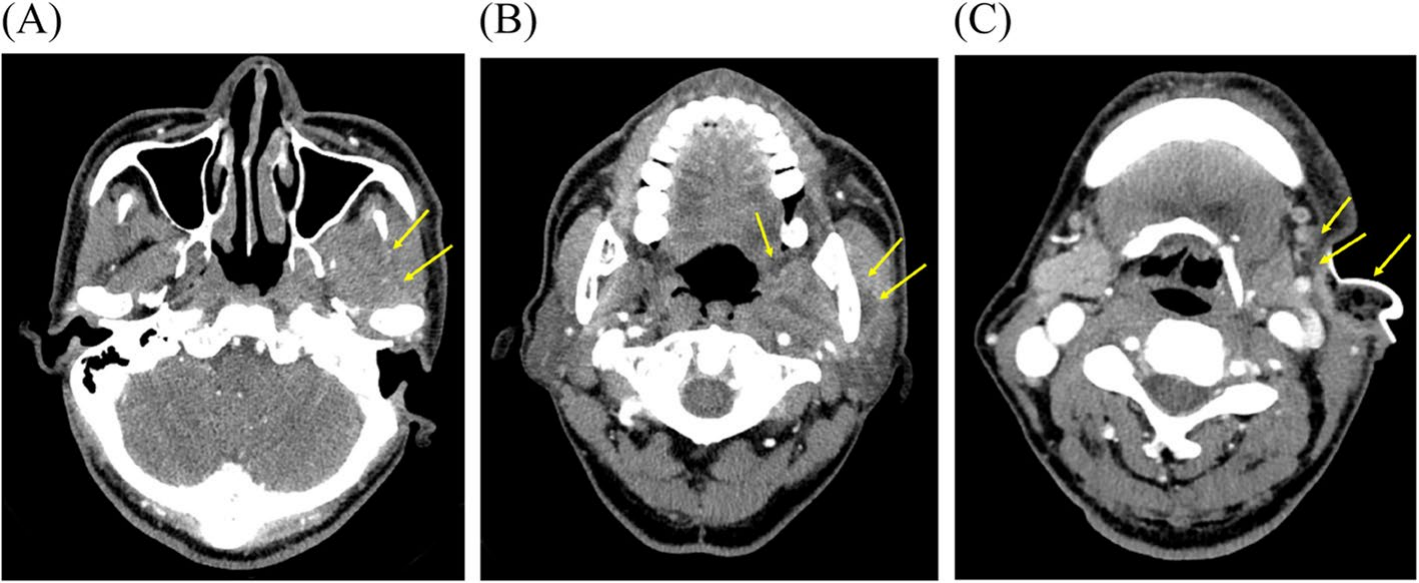
Neck angiography CT images 1 month after the first visit (case 2). Heterogeneous lesions (yellow arrows) were decreased but remained. **A** Masseteric space. **B** Parapharyngeal and masseteric space. **C** Submandibular space

A 65-year-old male patient from Seoul, Republic of Korea, visited the Department of Orofacial Pain and Oral Medicine at the Kyung Hee University Dental Hospital with the chief complaints of limited mouth opening and pain in the left TMJ for 10 days. During clinical examinations, his maximum mouth opening amount was only 10 mm, with pain in the left TMJ during mouth opening. During the lateral movement of the mandible, he exhibited a 5 mm range of movement to both the right and left sides. Palpation provoked pain on his left masseter and temporalis. An indistinct cortical outline of the left condyle was observed in the panoramic and TMJ series images. There were also findings of periodontal disease with vertical bone loss and furcation involvement in left molars. Since he had a history of rheumatoid arthritis of the hand, the initial diagnosis was osteoarthritis or rheumatoid arthritis of the left TMJ. We prescribed anti-inflammatories and muscle relaxants and planned to take cone-beam computed tomography and bone scan for further evaluation of arthritis. However, 5 days after the first visit, his pain had worsened, and severe swelling of his left face was observed. We decided to refer him to the Department of Oral and Maxillofacial Surgery for further evaluation. He was admitted to the ward and performed extra-oral incision and drainage (I&D), pus culture, neck angiography CT and blood test. In pus culture, *prevotella intermedia* and *olsenella uli* were detected, which are the members of the oral microbiome. In the blood test, signs of infection were observed: WBC 43660/μL, ESR 120 mm/hour, CRP 38.73 mg/dL. In the neck angiography CT, extensive abscess formation was observed spanning the left masticator, submandibular, parapharyngeal, and cervical spaces. The suspected source of infection was periodontal disease associated with alveolar bone loss in the left upper and lower molars (Fig. 3). They kept intravenous antibiotics and regular I&D. After 3 weeks, his symptoms and abscess extent followed enhanced CT were improved, but necrotic fasciitis of left mandible was observed (Fig. 4). They applied negative-pressure wound therapy, and the skin lesion gradually improved. After 2 months, he complained of no pain and facial swelling, and the mouth opening amount was improved to 35 mm. We will keep regular follow-up checks.

### Case 3

**Fig. 5:**
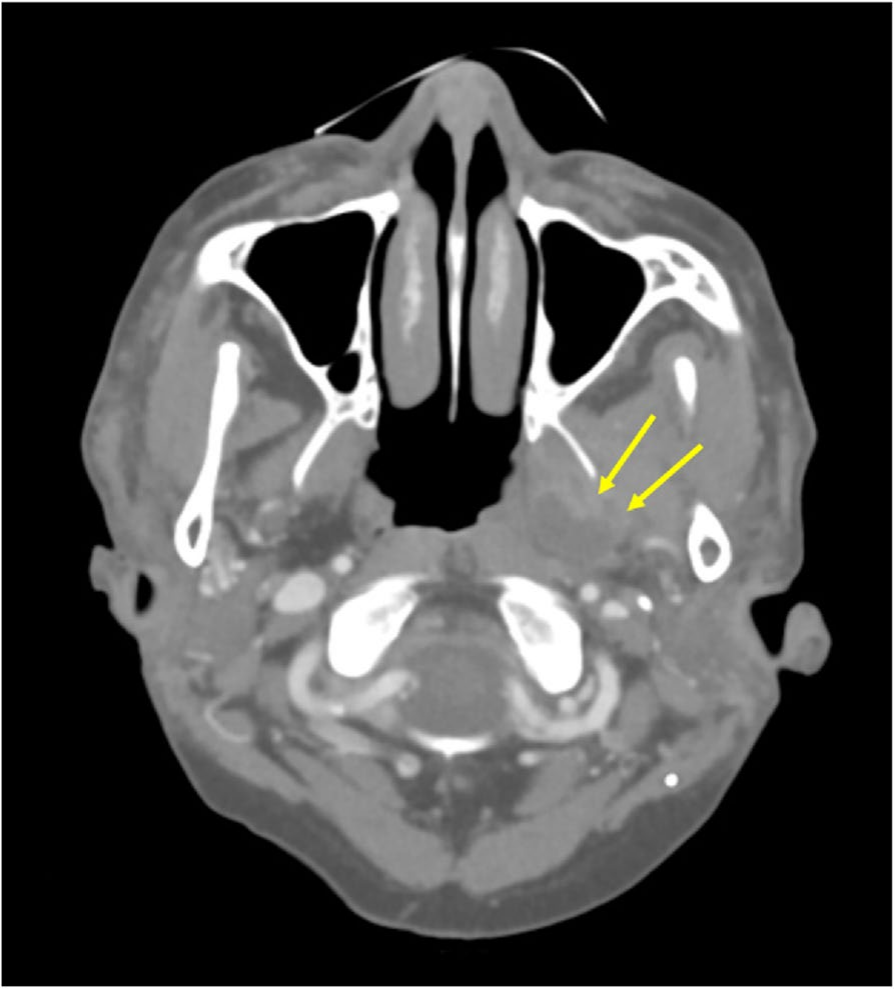
Enhanced CT image at 7 days after the first visit (case 3). Heterogeneous lesions of the left pterygoid and parapharyngeal space (yellow arrows) were observed

**Fig. 6:**
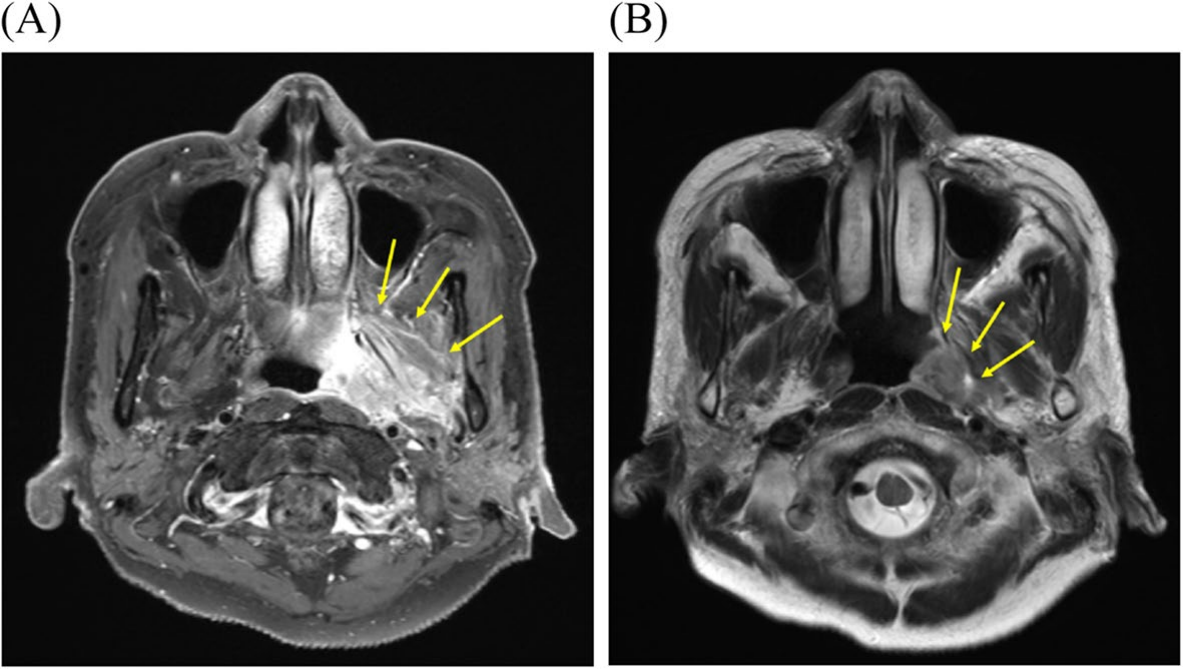
MRI images a month after the first visit (case 3). Increased signal reaction (yellow arrows) is observed in the left pterygoid and parapharyngeal area. **A** Triggered T1 image. **B** T2 image

A 67-year-old female patient from Seoul, Republic of Korea, visited the Department of Orofacial Pain and Oral Medicine at the Kyung Hee University Dental Hospital with the chief complaints of limited mouth opening and pain of left TMJ after getting scaling 1 month ago. In the panoramic image, alveolar bone loss with furcation involvement of #26 was observed, which was why she had scaled. During clinical examinations, her maximum mouth opening amount was only 14 mm, with pain in her left TMJ during mouth opening. During the lateral movement of the mandible, she exhibited a 3 mm movement to the right side and a 4 mm movement to the left side. She showed tenderness to palpation on the left TMJ and masseter, but facial swelling and local heat were not observed. The initial diagnosis was acute locking of the left TMJ and myalgia of the left masseter. We prescribed anti-inflammatories and muscle relaxants and performed physical therapy. However, 7 days after the first visit, her symptoms did not improve, and she complained of a foreign sensation in the throat. We suspected that there might be infectious lesions and decided to perform a blood test and enhanced CT. In the blood test, high-sensitivity CRP (hs-CRP) was 10.18 mg/dL, which suggests an infection. In the enhanced CT scan, abscess formation was observed in the left pterygoid and parapharyngeal spaces, with the suspected source of infection being periodontal disease associated with the #26 tooth (Fig. 5). We decided to start antibiotics and refer to the Department of Otolaryngology for further evaluation of parapharyngeal abscess. They suspected there might be hidden tumorous lesions, so they performed magnetic resonance imaging (MRI). In MRI, an increased signal of the pterygoid and parapharyngeal space was observed, which seemed to be an abscess rather than a mass (Fig. 6). They kept antibiotics for 2 months, and her symptoms were improved. There was no pain, and the maximum unassisted mouth opening was 40 mm. In the second blood test, hs-CRP was decreased to 0.23 mg/dL. After 4 months, she showed no recurrence, and there were no other symptoms.

Each patient showed similarities and differences in the clinical symptoms, diagnosis, and treatment courses in serial cases. A detailed comparison of each case is described (Tables 1 and 2). After undergoing serial treatment, all patients exhibited a decrease in tissue thickness assessed by CT or MRI images and showed improvement in the area of abscess lesions (Tables 3 and 4).

**Table 1:** Clinical characteristics and radiological findings of patients

	Case 1	Case 2	Case 3
**Duration from onset to first visit**	60 days	10 days	28 days
**Duration from first visit to abscess diagnosis**	Immediate	5 days	7 days
**Duration from onset to abscess diagnosis**	60 days	15 days	35 days
**Systemic comorbidity**	Hypertension Diabetes mellitus Dyslipidemia Hypothyroidism	Rheumatoid arthritis	None
**Diagnostic tool**	Blood test Enhanced CT Neck angiography CT	Blood test Neck angiography CT Pus culture	Blood test Enhanced CT MRI
**Suspicious sources of orofacial infection on panoramic image**	Periapical lesion of #26	Periodontal disease with alveolar bone loss of #26–27, 36–37	Periodontal disease with alveolar bone loss of #26
**Abscess location on imaging**	Masseteric space Temporal space	Masticator space Submandibular space Parapharyngeal space Cervical space	Parapharyngeal space Pterygoid space
**Treatment**	Antibiotics Admission	Antibiotics Extra-oral I&D Admission	Antibiotics
**Duration from abscess diagnosis to symptom improvement**	124 days	118 days	50 days

*CT* Computed tomography, *MRI* Magnetic resonance imaging, *I&D* Incision and drainage

**Table 2:** Common and individual characteristics of patients

**Common features**		
**Case 1, 2, and 3**	**Clinical**	Severe trismus; Maximum mouth opening under 15 mm Restricted range of lateral movement of the mandible (< 7 mm) Severe pain of orofacial area including masseter and TMJ (VAS > 7) Diagnosed with TMD at first visit
	**Hematological**	Increased WBC, CRP
	**Radiological**	Presence of suspicious sources of odontogenic infection Abscess formation observed in enhanced CT
**Case 1 and 2**	Facial swelling	
**Individual features**		
**Case 1**	Reoccurrence of abscess in masseteric, temporal space	
**Case 2**	Dyspnea due to parapharyngeal swelling Necrotizing fasciitis	
**Case 3**	No significant facial swelling & redness Dysphagia	

*TMJ* Temporomandibular joint, *VAS* Visual analog scale, *TMD* Temporomandibular disorder, *DC/TMD* Diagnostic criteria for TMD, *WBC* White blood cell *CRP* C-reactive protein

**Table 3:**
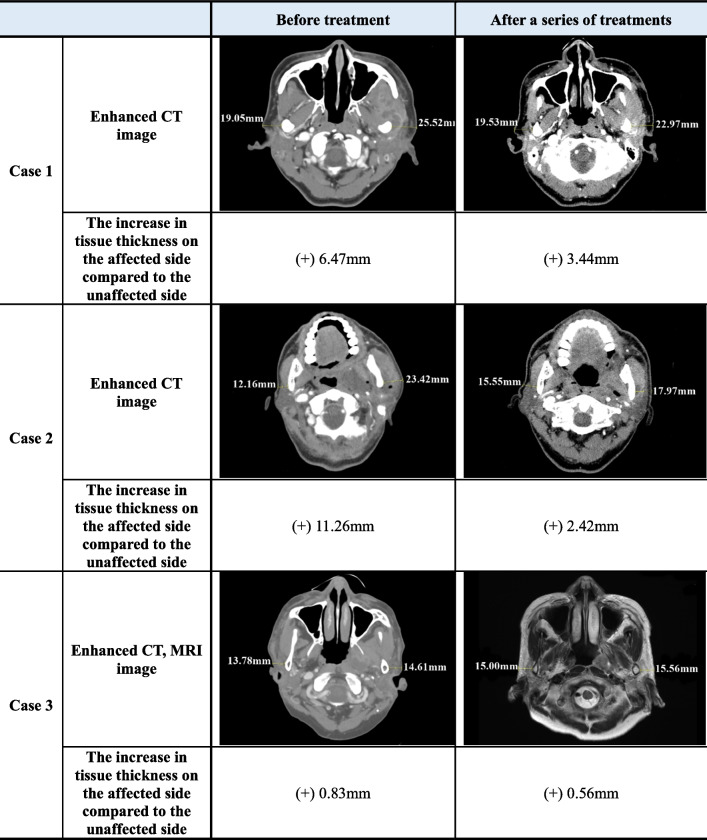
Difference in muscle thickness between affected and unaffected sides: at diagnosis and post-treatment

*CT* Computed tomography, *MRI* Magnetic resonance imaging

**Table 4:**
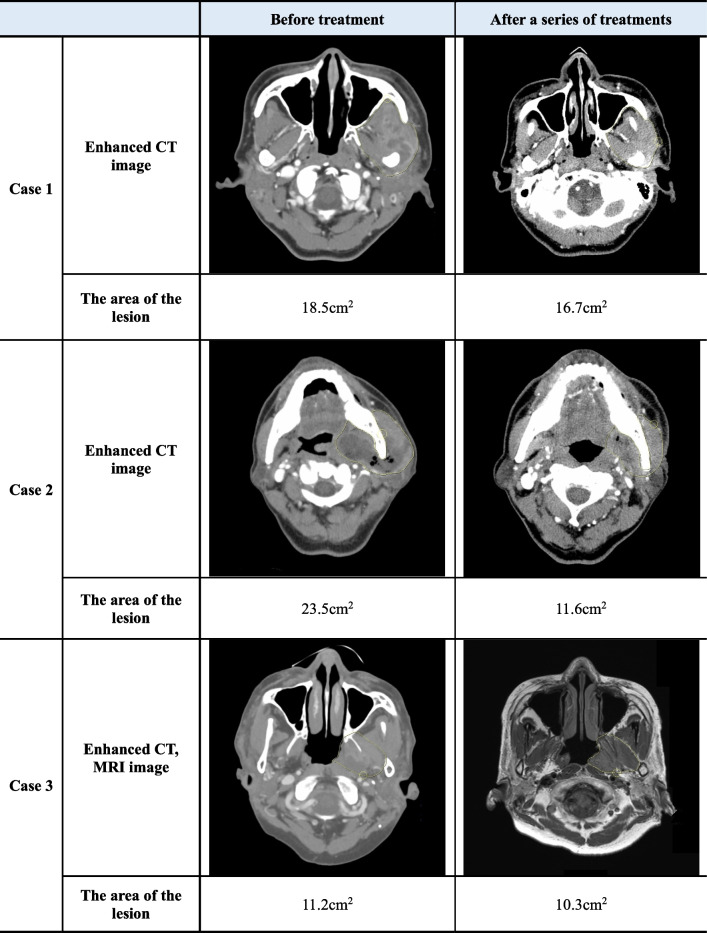
Measurement of lesion area in imaging: at diagnosis and post-treatment

*CT* Computed tomography, *MRI* Magnetic resonance imaging

## Discussion and conclusions

In these cases, we could identify patients initially diagnosed with TMD but finally proven to have fascial space abscesses. All patients complained of severely limited mouth opening with the pain of TMJ or masticatory muscles, similar to TMD symptoms. Like other infections, clinical signs and symptoms of orofacial fascial space are broad depending on the degree of infection. If there are only a few common symptoms, such as pain in the facial area and limited mouth opening, clinicians might neglect the possibility of space abscess.

Previous case series have reported the possibilities of fascial space abscess in the orofacial area (Table 5) [3, 9–27]. Of the 24 cases, 9 (37.5%) reported that distinct facial swelling was not observed at the first visit. Thus, patients were initially diagnosed with other diseases rather than space abscess: TMD, parotitis, headache and otitis media [3, 9, 13, 14, 18, 21, 26]. What made clinicians realize the possibility of a fascial space abscess were clinical symptoms, symptoms that were aggravated or not responded to treatment, and, additionally, symptoms such as facial swelling that strongly suggest a fascial space abscess.

**Table 5:** Summary of previous case reports of orofacial fascial space abscess

Case number, year [ref]	Age/sex	Clinical symptoms (Initial/ Additional)	Initial diagnosis	Diagnostic methods	Abscess location (space)	Suspicious sources of orofacial infection	Treatment	Duration from onset to first visit	Duration from first visit to abscess diagnosis	Duration from onset to abscess diagnosis
**1, 1996** **[**6**]**	62/M	Facial swelling, trismus, pain	Parotitis (at another clinic)	Enhanced CT, MRI, blood test	Masticator	#17; dental caries	Masseter muscle dissection, I&D, antibiotics	3 years	Immediate	3 years
**2, 1996** **[**7**]**	69/M	Facial swelling, dysphagia, pain, fever, tachypnea, tachycardia	Space abscess	CT, blood test	Masticator, submandibular, parapharyngeal, peritonsillar	#37; dental caries, periapical abscess	I&D, tracheotomy, antibiotics	Un-known	Immediate	Unknown
**3, 1999** **[**8**]**	51/M	Facial swelling, trismus, pain, fever	Space abscess	Panoramic view, ultrasound scan, blood test, urea test	Masticator	#38; pericoronitis	Needle aspiration, I&D, antibiotics	2 days	Immediate	2 days
**4, 1999** **[**8**]**	21/M	Facial swelling, dysphagia, trismus, lymphadenopathy	Space abscess	Panoramic view, ultrasound scan, blood test, urea test	Masticator	#38; pericoronitis	Needle aspiration, I&D, antibiotics	Unknown	Immediate	Unknown
**5, 2001** **[**9**]**	25/M	Facial swelling, pain, intra-oral fistula	Space abscess	Enhanced CT, CT, dual-isotope scan	Masticator, subdural	Right molars; dental caries, periapical abscess	I&D, antibiotics, neuro-surgical drainage	5 days	Immediate	5 days
**6, 2008** **[**10**]**	62/F	Trismus, pain	TMD (ADD without reduction)	MRI, blood test	Masticator	#17; periodontitis	I&D, antibiotics	1 week	5 days	12 days
**7, 2008** **[**10**]**	68/F	Trismus, pain	TMD (at another clinic)	Enhanced CT, MRI, blood test	Masticator	#37; dental caries, #38; pericoronitis	I&D, antibiotics	Unknown	Immediate	Unknown
**8, 2009** **[**11**]**	61/M	Headache, dysphagia, fever / Facial swelling, trismus	Headache	CT, blood test	Masticator, parapharyngeal, intra-orbital, intra-cranial	#26–27	I&D, bur hole drainage, antibiotics	1 week	5 days	12 days
**9, 2010** **[**12**]**	49/M	Facial swelling, trismus, pain, visual disturbance	Space abscess, thrombosis	CT, MRI, blood test	Masticator, parapharyngeal, cavernous sinus	#16,18,48; dental caries	I&D, antibiotics	5 days	Immediate	5 days
**10, 2011** **[**13**]**	50/F	Facial swelling, trismus, pain, fever, dyspnea, dysphagia, foreign sensation of neck	Space abscess	Panoramic view, CT, blood test	Masticator, submandibular, cervical	#45–47; dental caries	I&D, cervicotomy, antibiotics	10 days	Immediate	10 days
**11, 2013** **[**14**]**	28/F	Facial swelling, trismus, pain	Space abscess	CT	Masticator	#38; dental caries	I&D, antibiotics	1 week	Immediate	1 week
**12, 2013** **[**15**]**	6/M	Trismus, ear pain, fever, vomiting, neck stiffness	Otitis media (at another clinic)	CT, blood test	Masticator, TMJ space	Unknown	Arthrocentesis, antibiotics	1 week	Immediate	1 week
**12, 2013** **[**15**]**	18/F	Trismus, pain / Facial swelling	TMD (at another clinic)	Enhanced CT, blood test	Masticator, deep temporal, skull base, TMJ space	Left wisdom teeth	I&D, antibiotics, arthrocentesis	3 weeks	4 days	25 days
**13, 2014** **[**16**]**	14/F	Facial swelling, sinus congestion, gray drainage	Allergic fungal sinusitis	CT, MRI	Infratemporal, maxillary sinus, middle cranial	Sinus infection	Abscess drainage, craniotomy, antibiotics	1 year	Immediate	1 year
**14, 2014** **[**17**]**	75/M	Trismus, tonsillar swelling, pain / Facial swelling	Peritonsil abscess	CT, blood test	Masticator, submandibular, parapharyngeal	Peritonsilar abscess	I&D, antibiotics	5 days	2 days	1 week
**15, 2014** **[**17**]**	90/F	Facial swelling, trismus, pain	Peritonsil abscess	Enhanced CT, blood test	Masticator, parapharyngeal	Peritonsilar abscess	I&D, antibiotics	3 days	4 days	1 week
**16,2015** **[**18**]**	56/F	TMJ area swelling, trismus, pain, fever	TMD (at another clinic)	Ultrasound, MRI, blood test	Masticator, intracranial	Unknown	Antibiotics	1 week	Immediate	1 week
**17, 2015** **[**3**]**	66/M	Pain / Trismus	TMD	Enhanced CT, blood test	Masticator	Facial acupressure massage	I&D, antibiotics	3 days	1 week	10 days
**18, 2016** **[**19**]**	79/F	Facial swelling, trismus, pain	Space abscess	Enhanced CT	Masticator, parapharyngeal	Unknown	I&D, antibiotics	1 week	Immediate	1 week
**19, 2019** **[**20**]**	38	Facial swelling, pain, headache, oral ulcer	Space abscess	CT, MRI, blood test	Masticator, epidural	Lung infection	Burr hole drainage, antibiotics	3 weeks	Immediate	3 weeks
**20, 2020** **[**21**]**	53/M	Facial swelling, pain, fever	Space abscess	CT, MRI, blood test	Masticator	#38; pericoronitis	I&D, antibiotics	10 days	Immediate	10 days
**21, 2021** **[**22**]**	53/M	Facial swelling, pain, trismus, pain, speaking problem	Space abscess	Enhanced CT, blood test	Masticator, buccal, sphenoid bone, whole brain	#37; periodontitis	Neuro-surgical drainage, antibiotics	2 weeks	Immediate	2 weeks
**22, 2022** **[**23**]**	50s/M	Trismus, pain / Facial swelling, dysphagia	TMD (at another clinic)	Enhanced CT, blood test	Masticator, parotid, submandibular, parapharyngeal	Intra-muscular stimulation	I&D, antibiotics	4 weeks	Immediate	4 weeks
**23, 2023** **[**24**]**	84/F	Facial swelling, trismus, pain	Space abscess	CT	Masticator, infratemporal, extra-cranial	Unknown	I&D, antibiotics	2 days	Immediate	2 days

*M* Male, *F* Female, *CT* Computed tomography, *MRI* Magnetic resonance imaging, *I&D* Incision and drainage, *TMD* Temporomandibular disorder, *ADD* Anterior disc displacement, *TMJ* Temporomandibular joint

Meanwhile, 20 cases reported suspicious sources of infection (Table 5). In 14 cases (70%), the source of infection was odontogenic origin: pericoronitis, periodontitis, dental caries, and periapical lesion [9–18, 24, 25]. Among the odontogenic origins, maxillary molars were in 6 cases, and mandibular molars were in 11 cases. In 4 cases (20%), the source of infection was the spread of infection from other sites: sinus infection and peritonsillar abscess [19, 20, 23]. In 2 cases (10%), the infection source was invasive facial area treatments such as acupressure massage and intra-muscular stimulation [3, 26]. Once space abscess was suspicious, all cases used blood tests and additional imaging techniques to evaluate soft tissue, such as enhanced CT, MRI, or ultrasound, to confirm space abscess.

The clinical key to distinguishing hidden fascial space abscesses is to catch the possibility of infection. If distinct symptoms such as facial swelling, redness or fever are observed, it is reasonable to suspect a fascial space abscess. However, facial swelling or fever alone represents a non-specific symptom and cannot solely serve as a clear basis for diagnosing a fascial space abscess. Some fascial space abscesses can exist without evident facial swelling, and various diseases within the orofacial area, such as giant cell arteritis or autoimmune disorders like systemic lupus erythematosus, can mimic the symptoms of a fascial space abscess [28, 29]. From this perspective, clinical symptoms to suspect fascial space abscess should be severe mouth opening limitation, considering that limited mouth opening was observed in all 24 cases of fascial space abscess. Limited mouth opening in the orofacial fascial space abscess is because the inflammatory state of masticatory muscles and fascia induced the weakness and limited functions of masticatory muscles [30]; thus, limited mouth opening can represent the possibility of fascial space abscess in the orofacial area. Alongside limitations in vertical mouth opening, suspicion of orofacial fascial space abscess can arise when the mandibular movement during lateral excursion is less than 5 mm, as demonstrated in this case series.

If clinicians realize the possibility of a fascial space abscess, the next step is to search for possible sources of infections, which include recent infections of other regions, trauma, surgical treatment, or intravenous drug use [1, 31]. There might be systemic risk factors such as diabetes mellitus, steroid therapy, chemotherapy, and immune dysfunction. However, the most common cause of orofacial fascial space abscess is still odontogenic infection. Common origins of orofacial infection include dental caries, periapical lesions, inappropriate fillings, inadequate root canal treatment, pericoronitis, and periodontal disease [32]. As the treatment of odontogenic infection is delayed, the risk of spreading infection increases. Delayed treatment can result from diagnostic errors, patient disagreement with treatment, or socioeconomic factors among patients. In a recent study, individuals with a lower socioeconomic status exhibited a higher prevalence of untreated dental caries and poorer oral hygiene [33]. In case 1, the patient, a recipient of a medical aid program, took 60 days from the onset of symptoms to the first hospital visit. It was the longest duration among the three cases, and her socio-economic status might explain the reason behind this delay.

When the origins of orofacial infections are maxillary molars, infections mainly spread through the thin maxillary buccal plates, involving temporalis, lateral pterygoid, and masseter muscles [32, 34]. Since maxillary infections are less prone to spread downward, fascial space abscess with maxillary infection usually involves masticator, buccal, and parapharyngeal space. On the other hand, orofacial infections from mandibular molars show different patterns. Mandibular molar infections frequently spread into the masseter and medial pterygoid muscles and can involve lateral pterygoid or temporalis less frequently [34]. Eventually, mandibular infections mostly progress to masticator space abscesses and spread downward to form submandibular and sublingual space abscesses [7].

Among the possible infection pathways of odontogenic infections, isolated lateral pterygoid and parapharyngeal space abscesses are relatively uncommon but have been reported steadily, such as case 3 in this report [35]. The common pathway of parapharyngeal space abscess due to odontogenic infection is through masticator space; thus, maxillary and mandibular molars are both responsible for abscess formation [36]. However, when a parapharyngeal space abscess originates solely from an infection in the pterygoid space due to odontogenic causes, distinct facial swelling might not be observed [13, 37]. In cases of isolated pterygoid and parapharyngeal space abscess, odontogenic infection is more likely to stem from maxillary molars than mandibular molars.

Once clinicians suspect fascial space abscess by clinical symptoms and search for possible sources of infection, additional examinations are needed to confirm the abscess. A blood test is a simple but surely effective measurement to detect and estimate infectious lesions. Especially, some blood markers have been reported to show high sensitivity to infection. CRP is a broadly used blood marker, and it can effectively reflect the severity of infections, including fascial space abscesses [38, 39]. Since CRP decreases corresponding to the cure of infections, it can also help to evaluate the treatment efficacy and adjust the treatment plan efficiently. Meanwhile, hs-CRP is commonly used to evaluate cardiovascular disease, but it can also be used to detect fascial space abscesses, considering that hs-CRP reflects the inflammatory state of muscles and vessels [40, 41]. Compared with CRP, hs-CRP shows high sensitivity and can detect levels as low as 0.1 mg/dL; thus, it can more sensitively evaluate minor risk factors and course of treatment. Another useful biomarker is the neutrophil-to-lymphocyte ratio (NLR). Increased NLR is a state of increased neutrophils and decreased lymphocytes, which reflects an inflammatory state [42]. NLR is a simple biomarker that can be calculated from conventional differential counts, but it can evaluate not only the inflammatory state but also host immunity, which can determine the prognosis of fascial space abscess.

Imaging techniques are also needed to confirm the location and extent of abscess. Since fascial space abscess is a soft tissue disease, conventional imaging, such as panoramic images, cannot effectively detect fascial space abscess. Broadly used imaging techniques are enhanced CT and MRI. Both can reflect the actual state of abscess precisely and can identify the pathway of infection [7, 43]. Also, ultrasonography can be used to diagnose fascial space abscesses. In ultrasonography, an abscess shows a typical anechoic area, whereas inflammatory structures show a hyperechoic area [4]. Ultrasonography is a minimally invasive diagnostic tool known for its effectiveness in diagnosing soft tissue structures. Additionally, it can enhance the precision of invasive treatments like muscle injections or arthrocentesis [44]. Compared to MRI or enhanced CT, ultrasonography is slightly inferior in detecting the extent of fascial space infections, and it cannot detect deep space abscesses such as parapharyngeal abscesses [4, 45]. However, the key advantage of ultrasonography in diagnosing abscesses lies in its ability to assess the real-time condition of the abscess promptly, facilitating the swift formulation of additional diagnostic plans.

Once a fascial space abscess is definitively diagnosed, treatment should prioritize infection control. Alongside conventional methods like antibiotics or incision and drainage (I&D), several studies have introduced novel approaches. A local chemotherapeutic approach involves delivering antibiotics directly to the infection site, minimizing systemic complications. Specifically, in odontogenic infections, membrane or gel-type polymers—such as cellulose or polysaccharides—containing dental drugs can be targeted to specific areas in the oral cavity, such as periodontal tissues with significant pocket depth and alveolar bone loss [46, 47]. As a more conservative therapy, low-level laser therapy has been suggested for its potential anti-inflammatory effects in focal lesions. However, its efficacy in infection control has not yet been established [48].

When dealing with an orofacial fascial space abscess, early recognition of the potential for a space abscess is crucial. This can be attained by recognizing clinical symptoms such as restricted mouth opening accompanied by facial pain, including discomfort in the TMJ or masticatory muscles, particularly when these symptoms have recently emerged. In this case series, limitations were noted in both the vertical movement and lateral excursion of the mandible when compared to the normal range. When assessing mandibular motion in units of millimeters (mm), restriction is indicated in the side-to-side lateral excursion when it measures less than 7 mm, while the mouth opening is < 35 mm [49, 50]. The presence of limitations in vertical and side-to-side mouth opening is important in discerning orofacial fascial space abscess. However, clinicians must recognize that various conditions can mimic both TMD and orofacial fascial space abscess. Therefore, the next crucial step in diagnosing orofacial fascial space abscess should involve investigating potential sources of infection. Since the delayed intervention of orofacial fascial space abscess can cause increased morbidity and mortality, clinicians must also consider the possibility of fascial space abscess in TMD patients to prevent unexpected progress of orofacial abscess.

## Data Availability

The datasets used and/or analyzed in the current study are available from the corresponding author upon reasonable request.

## Abbreviations

CRP: C-reactive protein
CT: Computed tomography
ESR: Erythrocyte sedimentation rate
hs-CRP: High-sensitive C-reactive protein
I&D: Incision and drainage
MRI: Magnetic resonance imaging
NLR: Neutrophil-to-lymphocyte ratio
TMD: Temporomandibular disorder
TMJ: Temporomandibular joint
WBC: White blood cell
mm: Millimeter
